# What is the optimal gestational age for twin delivery

**DOI:** 10.1186/1471-2393-6-3

**Published:** 2006-02-16

**Authors:** Ahmad F Bakr, Tarek Karkour

**Affiliations:** 1 Department of Pediatrics, University of Alexandria, Alexandria, Egypt; 2 Department of Obstetrics, University of Alexandria, Alexandria, Egypt; 3 Consultant Neonatologist, Welcare Hospital, PO Box 31500 Dubai, United Arab Emirates

## Abstract

**Background:**

The question about outcome in twins delivered early versus late remains unanswered. The *objective *of this study was to evaluate the association of the timing of delivering twins and the perinatal outcome.

**Methods:**

A prospective cohort study was carried-out in Alexandria University Maternity Hospital. We planned to examine the records of twin deliveries over 2 years. The inclusion criteria were twin deliveries with gestational age at delivery at least 36 completed weeks. Twins of mothers with chronic illness and those with congenital anomalies were excluded. Perinatal outcome parameters (morbidity and mortality) were defined and evaluated.

**Results:**

Out of 273 twin sets, 197 (72.2%) met the inclusion criteria. They were classified into 3 groups according to the gestational age at delivery. Neonatal morbidity and maternal complications were higher in those delivered earlier. Twins electively delivered had worse outcome than those delivered spontaneously. In the elective group, there was no difference in the outcome between those delivered earlier or later.

**Conclusion:**

Twins, when the pregnancy is uncomplicated, continue to grow and mature with advancement of the gestational age. In the absence of significant maternal complications, it is advisable to deliver twins only at 38 completed weeks' gestation or later to avoid neonatal complications.

## Background

Lots of controversies are raised about the optimal gestational age for twin delivery. Whether multiple pregnancy has an adverse effect or not on fetal lung maturity remains very debatable. Luke et al [1] suggested that twins attain more rapid pulmonary maturation than singletons and therefore complications of postmaturity may arise sooner in twins than in singletons. This has been also ascertained by the study of Leveno et al [2], and referred to in the recommendations of the American College of Obstetricians and Gynecologists since 1989 [3]. This has resulted in a tendency to electively deliver twins earlier than singletons of comparable gestational age. Hack et al have found an association between elective delivery and adverse perinatal outcome, in particular respiratory morbidity [4]. This was also reported by Wax et al in 2002 [5]. Friedman et al[6] recently reported that twins do not have accelerated maturation compared with singletons for the same gestational age.

Complications related to twin pregnancy and delivery are enormous. Our local statistics point to an incidence of 1.4% for twin gestations. With the recent introduction of assisted reproduction techniques, the number seems to be increasing. Perinatal mortality is known to be approximately seven times higher in twins than singletons [8]. Many of these complications are related to preterm delivery, low birth weight, and respiratory problems [9]. This issue is furthermore complicated in developing countries, where the level of neonatal intensive care is not optimal. The fact that, complications of prematurity and low birth weights are more common in these set-ups,[10] further complicates the decision.

The question about outcome in twins delivered early versus late remains unanswered [11]. However, a better understanding of the relationship between timing of delivery and perinatal outcome in local set-ups could be used to decrease the morbidity, mortality associated with twin deliveries [12]. The purpose of this study was to evaluate the association of the timing of delivering twins and the outcome. We planned a study to test the perinatal outcome of twin pregnancies delivered at 36–37 weeks' gestation and twin pregnancies delivered at ≥ 38 weeks' gestation.

## Methods

This observational cohort study was carried-out at El-Chatby Maternity Hospital, University of Alexandria, Egypt. El-Chatby hospital is a busy maternity hospital with a large number of deliveries approaching 10,000 per year. Twin deliveries between January 1, 2000 and December 31, 2002, with gestational age at delivery at least 36 completed weeks, were included. Exclusion criteria were significant maternal chronic ill health not related to pregnancy, twin-twin transfusion syndrome, congenital anomalies and the death of one fetus. The protocol was approved by the University board.

Patient information was abstracted from the maternal and infants' charts. Maternal data (last menstrual period, delivery indication and the presence of complications as oligohydramnios, preeclampsia, fetal distress, placental abruption or others) and newborn data (gestational age, birth weight, Apgar score, need for resuscitation, need for intensive care admission and clinical course if admitted i.e. complications and outcome) were collected. Respiratory distress syndrome was diagnosed if respiratory distress was associated with the classic X-ray findings. Hypoglycemia was considered if random blood glucose level was < 40 mg/dl. The diagnosis of neonatal sepsis necessitated a positive blood culture in the presence of suggestive clinical signs. Oligohydramnios was determined by sonographic examination (amniotic fluid index of ≤ 5) [13]. Maternal hypertension with proteinuria or edema were used to diagnose preeclampsia. Fetal distress, antepartum or intrapartum, was defined according to the international standards [14]. Truly elective deliveries were defined as those that were not spontaneous or complicated with preeclampsia, oligohydramnios, fetal growth restriction (FGR) or abruption. Gestational age at delivery was defined as the number of completed weeks from the first day of the last normal menstrual period to the date of delivery. Early ultrasound examination (less than 20 weeks) results were used if the dates were unreliable. FGR was considered if the fetal weight is below the 10th percentile of gestational age [15].

Perinatal outcomes were compared between twin gestation delivered at < 38 weeks' gestation and those delivered at ≥ 38 weeks' gestation. Chi-square test student's *t*-test and were used for the analysis. *P *values < 0.05 were considered significant.

## Results

During the 2-year study period, there were 18794 deliveries and 273 twin sets (1.45%). Out of the 273 sets, 197 (72.2%) met the inclusion criteria. Classified according to the gestational age at delivery, 115 sets (58.4%) were born at 36–<37 weeks[Group I], 66 (33.5%) at 37–<39 weeks [Group II] and 16 (8.1%) at > 39 weeks [Group III].

Table 1 summarizes the data and the perinatal outcome of these three groups. Lower birth weight, and the need for neonatal intensive care were significantly present in groups I and II compared to group III. FGR was pronounced in group I compared to group III. Respiratory distress, sepsis and hypoglycemia were more frequently encountered in newborns of group I. In group II, their incidence was comparable to that of group III. Maternal complications (preeclampsia, oligohydramnios, fetal distress and placental abruption) were significantly higher in group I than group III. Though their incidence in group II was higher than in group III, yet the difference was not significant.

**Table 1:** Perinatal outcome according to the gestational age

	**Group I **36–<37 Wks (115 sets)	**Group II **37–<39 Wks (66 sets)	**Group III **> 39 Wks (16 sets)	***p *value**	
**Newborn Data**				**Comparison**	
Number of Newborns	**230**	**132**	**32**	I ***Vs ***III	II ***Vs ***III
Birth weight (mean ± SD) g	2494 ±278	2799 ±217	2983 ±246	***S***	***S***
Apgar score at 5 minutes (mode)	8	9	10	***S***	***NS***
Fetal Growth Restriction	38 (16.5%)	8 (6.1%)	2 (6.3%)	***S***	***NS***
Neonatal intensive care required	26 (11.3%)	6 (4.5%)	0 (0%)	***S***	***S***
Respiratory distress syndrome	16 (7%)	1 (0.8%)	0 (0%)	***S***	***NS***
Sepsis	12 (5.2%)	1 (0.8%)	0 (0%)	***S***	***NS***
Hypoglycemia	5 (2.2%)	0 (0%)	0 (0%)	***S***	***NS***
**Maternal Data**					
Number	**115**	**66**	**16**	I ***Vs ***III	II ***Vs ***III
Preeclampsia	8 (7%)	1 (1.5%)	(0%)	***S***	***NS***
Oligohydamnios	11 (9.6%)	1 (1.5%)	0 (0%)	***S***	***NS***
Antepartum fetal distress	4 (3.5%)	2 (3%)	0(0%)	***S***	***S***
Placental abruption	3 (2.6%)	0 (0%)	0 (0%)	***S***	***NS***

***S ***= significant, *P *< 0.05 ***NS ***= non-significant, *P *> 0.05

Eighty-eight sets have undergone elective deliveries for various causes. Table 2 summarizes the perinatal outcome of the twin sets according to the type of delivery (elective versus spontaneous). The birth weights and Apgar score were comparable, but FGR, the need for intensive care and the occurrence of respiratory distress syndrome were significantly higher in the newborns of the elective group. All the outcome parameters were significantly higher in mothers of the elective group. When the elective group was classified by gestational age into early and late deliveries, no significant difference was found in all the perinatal outcome parameters. (Table 3).

**Table 2:** Perinatal outcome according to the type of delivery

	**Elective Group **(88 sets)	**Spontaneous Group **(109 sets)	***p *value**
**Newborn Data**			
Number of Newborns	176	218	**Comparison**
Birth weight (mean ±SD) g	2691 ±274	2759 ±223	***NS***
Apgar score at 5 minutes (mode)	8	9	***NS***
Fetal Growth Restriction	26 (14.6%)	8 (3.7%)	***S***
Neonatal intensive care required	23 (13.1%)	11 (5%)	***S***
Respiratory distress syndrome	13 (7.3%)	4 (1.8%)	***S***
Sepsis	9 (5.1%)	7 (3.2%)	***NS***
Hypoglycemia	3 (1.7%)	2 (0.9%)	***NS***
**Maternal Data**			
Number	88	109	**Comparison**
Preeclampsia	7 (8%)	2 (1.8%)	***S***
Oligohydamnios	4 (4.5%)	1 (0.9%)	***S***
Antepartum fetal distress	5 (5.7%)	1 (0.9%)	***S***
Placental abruption	3 (3.4%)	0 (0%)	***S***

***S ***= significant, *P *< 0.05 ***NS ***= non-significant, *P *> 0.05

**Table 3:** Perinatal outcome among elective deliveries according to the gestational age

	**Elective, Early (36–37 Wks)**	**Elective, Late (> 38 Wks)**	***p *value**
**Newborn Data**			
Number of Newborns	92	84	**Comparison**
Birth weight (mean ±SD) g	2684 ±263	2762 ±251	***NS***
Apgar score at 5 minutes	8.6 ±0.89	8.6 ±0.93	***NS***
Fetal Growth Restriction	14 (15.2%)	12 (14.3%)	***NS***
Neonatal intensive care required	13(14.1%)	10 (12%)	***NS***
Respiratory distress syndrome	8 (8.7%)	5 (6%)	***NS***
Sepsis	5 (5.4%)	4 (4.8%)	***NS***
Hypoglycemia	1 (1.1%)	2 (2.4%)	***NS***
**Maternal Data**			
Number	46	42	**Comparison**
Preeclampsia	4 (8.7%)	3 (7.1%)	***NS***
Oligohydamnios	2 (4.3%)	2 (4.8%)	***NS***
Antepartum fetal distress	3 (6.5%)	2 (4.8%)	***NS***
Placental abruption	1 (2.1%)	2 (4.8%)	***NS***

***NS ***= non-significant, *P *> 0.05

## Discussion

The confusion, created by the belief that fetal lung maturity occurs several weeks earlier in twins compared with uncomplicated matched singletons [2], led to the widespread impression that twins mature sooner than the singletons counterpart. Moreover, the demonstration of advanced placental maturation in twins supported that impression [16]. All that encouraged the practice of delivering twins earlier. In our study, the incidence of neonatal respiratory distress syndrome was significantly higher at 36–37 weeks' gestation. It decreased and disappeared at higher gestational ages. Our findings go along with the findings of Friedman et al; who reported that twins < 36 weeks' gestation did not show accelerated maturation or improved neonatal outcome compared with their singletons counterpart [6]. The studies of Luke et al and Kiely et al suggested that Contrary FGR increases in twins after 38 weeks' gestation [1,17]. In our series, the incidence of FGR significantly decreased as the gestational age at delivery advanced. This might be explained by the fact that complicated pregnancies with FGR might need to be terminated earlier. Moreover, the mean birth weights increased steadily with advancement of the gestational age. Twins do continue to grow in utero till 40 weeks. The presence of higher maternal complications in group I (deliveries before 37 weeks) may, in part, explain the earlier termination of these pregnancies.

Comparing the electively delivered group with the spontaneously delivered group showed that the overall neonatal morbidity was worse in those delivered electively. This is in agreement with the findings that electively delivered newborns, even at term, have a higher incidence of severe respiratory distress and other complications [5,18,19]. The same was found for the maternal complications. Again this may explain the reasons for their elective delivery. When the elective deliveries were analyzed according to the gestational age at labor, no significant difference was found in the perinatal outcome parameters. Early and late elective twin deliveries had similar outcomes.

One of the limitations of our study was the design. A well-designed prospective randomized study involving all twin pregnancies and outcomes, at all the gestational ages will be more informative. Another limitation is the nature of our hospital. It is a tertiary referral centre which receives all complicated deliveries. This could make the sample not totally representative of the general population. Excluding twins of chronically ill mother is another issue. These may really experience advanced maturation.

## Conclusion

Twins, when the pregnancy is uncomplicated, continue to grow and mature with advancement of the gestational age. In the absence of significant maternal complications, it is advisable to deliver twins only at 38 completed weeks' gestation or later to avoid neonatal complications.

## Competing interests

The author(s) declare that they have no competing interests.

## Authors' contributions

AB carried out the study design, data collection, analysis and writing of the manuscript. TK shared participated in the design of the study and shared in the data collection. All authors read and approved the final manuscript.

